# Conduct problems, hyperactivity, and screen time among community youth: can mindfulness help? an exploratory study

**DOI:** 10.3389/fpsyt.2024.1248963

**Published:** 2024-04-09

**Authors:** Soyeon Kim, Stephanie Munten, Nathan J. Kolla, Barna Konkolÿ Thege

**Affiliations:** ^1^ Waypoint Research Institute, Waypoint Centre for Mental Health Care, Penetanguishene, ON, Canada; ^2^ Department of Psychiatry and Behavioural Neurosciences, McMaster University, Hamilton, ON, Canada; ^3^ Centre for Addiction and Mental Health, University of Toronto, Toronto, ON, Canada; ^4^ Department of Psychiatry, University of Toronto, Toronto, ON, Canada; ^5^ Department of Psychiatry, University of Saskatchewan, Saskatoon, SK, Canada

**Keywords:** screen time, youth, hyperactivity, conduct problems, mindfulness

## Abstract

**Background:**

The influence of mindfulness-based intervention (MBI) programs on behavioural problems among community youth is largely understudied. While technology continues to evolve and the prevalence of screen-based activities is rising, limited studies have accounted for screen time when examining the efficacy of an MBI. Accordingly, this study investigated the impact of MBI on conduct problems and hyperactivity among community youth, accounting for sociodemographic characteristics and four types of screen time.

**Method:**

Linear regression models were used to investigate 1) the association between four types of screen time and behavioural problems (i.e., conduct problems and hyperactivity) and 2) the efficacy of online mindfulness programs in reducing behavioural problems among community youth. The data were collected at baseline, intervention completion and 1-month follow-up (Spring 2021 to Spring 2022) in Ontario, Canada (*n*=117, mean age=16.82, male=22%, non-White=21%).

**Results:**

The average score for conduct problems was within the normal range, while the average score for hyperactivity was considered borderline at baseline. Accounting for other types of screen time, time spent playing video games was significantly associated with increased conduct problems (*β*= 1.75, *p*=.03), albeit rendering non-significant after correcting for multiple comparisons. The online mindfulness program was significantly associated with reduced hyperactivity, controlling for baseline mental health, age, sex and screen time.

**Conclusion:**

The current findings suggest a 12-week online mindfulness program may play a positive role in reducing hyperactivity even when accounting for screen time. Our findings advocate the evidence base on the efficacy of MBI in managing hyperactivity.

## 1 Introduction

The COVID-19 pandemic has significantly worsened the mental health of Western youth (1). In particular, research indicates that the COVID-19 pandemic and associated public health measures have disproportionately influenced the health and well-being of youth worldwide (2–4), mainly through increased sedentary behaviours, screen time, and adverse psychological effects (5–7). The apparent impact of increased screen time on mental well-being has heightened the need for support programs specifically aimed at improving youth mental health and wellbeing, including virtual mindfulness-based programs (8). However, whether support programs, such as mindfulness, can mitigate behavioural difficulties in community youth, such as conduct problems and hyperactivity, while accounting for increases in screen time, is vastly understudied and warrants further investigation (9).

Two common behavioural difficulties youth experience are conduct problems and hyperactivity. Conduct problems refer to youth engaging in more aggressive behaviours and disregarding others. Youth with higher screen time durations are more likely to engage in adverse behavioural conduct, as shown in longitudinal and cross-sectional studies (10). It is suggested that exposure to video games may be a risk factor for aggressive behaviour, particularly if the game does not have a prosocial component or elicit a competitive response (11, 12). In fact, increased time spent playing video games, as well as watching TV, demonstrated a strong association with behavioural difficulties: youth engaging in higher amounts of screen time were more likely to be aggressive or engage in risky behaviours (11, 13, 14). Hyperactivity is another common behavioural problem among youth. It is often referred to in the literature as an attention-deficit/hyperactivity disorder (ADHD)-related symptom. Previous literature has shown associations between adolescent hyperactivity and screen-based sedentary behaviour time, such as playing video games and watching TV (15). The severity of hyperactivity appears to correspond to overall screen time through dose-dependent associations, where high screen time is associated with an increased risk of hyperactivity problems (16, 17). However, one type of screen time that has yet to be fully accounted for in previous literature is handheld devices (i.e., smartphones), as much of the literature reports on data only collected up to 2013. While smartphone devices were available then, their use among youth only started to rise in the early 2010s (18). Smartphone technology provides easy access to screen-based activities and has increased screen multitasking (i.e., engaging in more than one activity simultaneously) (18, 19). While previous studies have shown the negative influence of smartphones on youth psychological well-being (18), there is limited knowledge on how behaviour problems could be influenced by multitasking and increased screen access that has been made easier with smartphones.

Behavioural difficulties among youth, including conduct problems and hyperactivity, can be treated through various types of non-medicinal interventions and behavioural training programs such as physical activity and behavioural training programs. Mindfulness-based practices have been shown to change brain activation patterns linked with attention and concentration, making them a therapy of interest for managing difficulties with impulse control and reactivity (20–23). As such, mindfulness-based interventions (MBIs) may be a good way to mitigate various behavioural problems in youth. Previous literature has mainly focused on the effects of MBIs on ADHD among adolescents and less on other behavioural issues, such as conduct problems. While many studies report reductions in hyperactivity following participation in mindfulness sessions by supervising adults and adolescents, there are many that found minimal benefit or have not shown any benefits at all (20, 22–26). The lack of consensus regarding the benefits of MBIs may be due to the length of the MBI intervention, where shorter interventions (<7 weekly interventions) are less likely to impact behaviour (20). As such, it is recommended that longer intervention durations are employed when targeting behavioural issues.

High levels of screen time are prevalent among adolescents and young adults, who report spending up to 10 hours per day on various screen types (17). While digital platforms have provided an avenue for socialization during gathering restrictions, increased screen time among youth has been associated with adverse mental health outcomes (27). Research before the pandemic highlighted strong evidence for the associations between high durations of screen time and mental health indicators, including poorer mental well-being and inattention problems (10, 28–30). With technology continuing to evolve and the prevalence of screen-based activities rising, limited studies have accounted for the differential effect of various types of screen time on youth behavioural issues. Further, the influence of mindfulness-based intervention programs on behavioural problems beyond hyperactivity is mainly unknown. Therefore, this study aimed to investigate whether participating in a 12-week mindfulness program can mitigate behavioural issues among community youth while accounting for the influence of screen time on behavioural problems. The current study responds to the critical need to combat mental wellness challenges exacerbated amid a global pandemic among community youth. This study aims to support the implementation of a mindfulness-based program delivered virtually among youth as we transition to a “new normal”.

## 2 Methods

### 2.1 Procedure and participants

**Figure 1:**
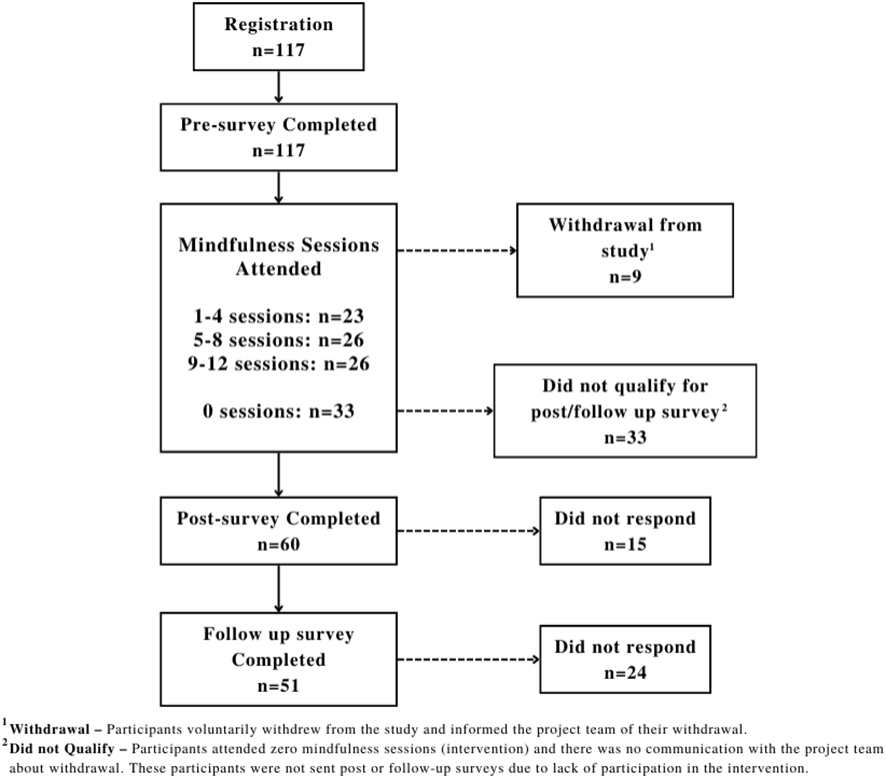
Flow diagram of participant attrition and pre-, post-, follow up surveys completion.

The present study is part of the “Mindfulness and Social-Emotional Learning in Youth” project, designed to examine the impact of a virtually-delivered mindfulness intervention on social-emotional competence in youth who engage in screen-based activities (31). The current study used the same sample to focus on the relationship between screen time and behavioural problems and the efficacy of mindfulness-based programs on behavioural problems (hyperactivity and conduct problems), while the previous study focused on the overall effectiveness of mindfulness-based programs on social-emotional competency (i.e., resilience, self-esteem, self-compassion) (31). Community youth aged 12-25 from Ontario, Canada, were recruited during the COVID-19 pandemic between April 2021 and April 2022 using digital flyers and word-of-mouth through partnering youth-serving organizations. **Figure 1** depicts the project timeline and participation rates throughout each time point. The final sample included 117 participants across five cohorts engaged in the intervention from spring 2021 to spring 2022. Participants, with an average age of 16.8 years (SD = 3.7; range 12-25), comprised 78.4% females. For those 15 year or younger at consent, parents received an informational letter. The pre-survey (n = 117; baseline) occurred before the 12-week intervention, followed by a post-survey (n = 60) immediately after completion of the intervention, and a follow-up survey (n = 51) one month later. Attendance in at least one mindfulness session was required for post- and follow-up survey eligibility. Participants who completed post and follow up surveys were offered a $25 gift card as a thank-you gift for their time. The institutional Research Ethics Board approved all components of this study (HPRA# 21.03.02).

### 2.2 Intervention

The Mindfulness Ambassador Program (MAP), developed by the non-profit organization Mindfulness Without Borders, is a structured 12-week, evidence-based group-mindfulness initiative (32–34). Participants attended weekly one-hour sessions, each focusing on a specific topic and incorporating social and emotional capacity-building practices like inspirational quotes, dialogue prompts, mindfulness exercises, and journaling. These elements fostered group discussions and learning, allowing space for individual reflection. The core of MAP’s curriculum lies in cultivating mindfulness skills to facilitate authentic discussions, emphasizing participation, self-reflection, critical thinking, and perspective-taking to enhance social and emotional competence. Theme of the MAP sessions are: session 1: mindfulness basics, session 2: paying attention, session 3: discovering inside, session 4: connecting authentically, session 5: practicing gratitude, session 6: mind-body connection, session 7: emotional intelligence, session 8: noticing emotional triggers, session 9: exploring open-mindedness, session 10: handling conflict skillfully, session 11: nurturing compassion, session 12: being the change. Two MAP-certified facilitators and one observer led all 12 sessions, conducted online through live Zoom meetings with a maximum of 20 youth participants. Weekly attendance was documented, and a fidelity checklist was completed after each session to ensure consistent program facilitation across cohorts.

### 2.3 Measures

#### 2.3.1 Demographics

Participant age and sex assigned at birth were was collected in a pre-survey and dichotomized as “adolescent” (12-17 years) or “young adult” (≥ 18 years) and “male” or “female,” respectively.

#### 2.3.2 Baseline mental health

At baseline, all participants are asked to complete a demographic questionnaire, including age, categorized as “adolescent” (12-17 years of age) or “young adult” (≥ 18 years of age), and sex assigned at birth, categorized as “male” or “female”. General mental health is measured using the Strengths and Difficulties Questionnaire [SDQ(S) 11-17 (35);], which is a 25-item self-report questionnaire consisting of five subscales, including emotional symptoms, conduct problems, hyperactivity, peer problems, and prosociality. The scale has demonstrated acceptable psychometric properties in youth populations in North America, with an internal consistency (Cronbach’s α) across subscales to be about 0.73 and acceptable test-retest reliability at 4 to 6 months (r=0.62) (36). Based on established literature cut-offs using the scale’s total score, the risk of participants having significant mental health problems can be categorized as: “low risk” (total scores =0-15), “moderate risk” (total scores = 16-19), or “high risk” (total scores = 20-40) (35).

#### 2.3.3 Behavioural problems

The Conduct Problems Subscale from the SDQ(S) 11-17 was one of the two tools selected to assess behavioural problems. The subscale consists of five items, displayed in **Table 1**, with one reverse-scored item. Scores for each item are summed to compute a single score of Conduct Problems (range 0-10), where higher scores indicate greater difficulties. Total scores ranging from 0-3 were coded as “low risk for significant problems,” 4 as “moderate risk for the significant problem,” and 5-10 as “high risk for significant problems” (37).

**Table 1:** Strength and Difficulties Questionnaire subscale items.

SDQ Subscale	Questionnaire items
Conduct Problems	1. I get very angry and often lose my temper. 2. I usually do as I am told. 3. I fight a lot. I can make other people do what I want. 4. I am often accused of lying or cheating. 5. I take things that are not mine from home, school or elsewhere.
Hyperactivity	1. I am restless, I cannot stay still for long. 2. I am constantly fidgeting or squirming. 3. I am easily distracted, I find it difficult to concentrate. 4. I think before I do things. 5. I finish the work I’m doing. My attention is good.

The Hyperactivity Subscale from the SDQ(S) 11-17 was the second tool selected to assess behavioural problems. The subscale consists of five items, displayed in **Table 1**, with two reverse-scored items. Scores for each item are summed to compute a single score of Hyperactivity (range 0-10), where higher scores indicate greater difficulties. Total scores ranging from 0-5 were coded as “low risk for significant problems,” 6 as “moderate risk for the significant problem,” and 7-10 as “high risk for significant problems” (37).

#### 2.3.4 Screen time

Participants were tasked with self-reporting their daily screen time, measured in hours, across the past seven days. Screen time categories encompassed video viewing (passively watching TV, movies, or videos), social media (including platforms like Facebook, Instagram, Snapchat), video games (both online and offline gameplay), and educational use (employing electronic devices like computers, laptops, or tablets for educational purposes). Quantification utilized a 4-point Likert scale, ranging from 1 to 4, with response options spanning less than 1 hour, 1-3 hours, 3-5 hours, and more than 5 hours for each specified screen type.

### 2.4 Analyses

The relationship between screen time and behavioural problems were examined using linear regression on baseline data. The potential impact of MAP on behavioural problems over the three time points (pre, post, and follow-up) were examined using Generalized Least Squares (GLS), an extension of generalized linear model suitable for longitudinal data with a cohort, where error terms of the same subject are likely correlated (38–40). We applied GLS via the Maximum Likelihood (ML) model to account for missing data and an unstructured model in consideration of the small sample size and less than 5 time points. In addition, a sensitivity analysis comparing age, sex, screen time, hyperactivity and conduct problems, and mental health status indicators at baseline between those who dropped out of the program and completed was conducted to determine if the drop-out was random. No significant difference between the dropouts and completers was found. Two sets of regression models were used to examine the efficacy of the mindfulness intervention on hyperactivity and conduct problems. First, we conducted a partially adjusted model accounting for participant characteristics (sex and age) and baseline mental health status [total SDQ(S) 11-17 score at baseline], examining the impact of a mindfulness intervention on hyperactivity and conduct problems. Then, a fully adjusted model accounting for four types of screen time (video viewing, social media, video games, and educational) was conducted to verify the additive impact of screen time in the association between the mindfulness intervention and bavioural problems. Lastly, to avoid spurious findings with inflated Type 1 error, we have adjusted the p-values for multiple tests using Holm’s method (41). The Holm’s method begins with the most significant p-value and iteratively moves towards the least significant p-value while adjusting for the significance threshold at each step. It is less conservative than Bonferroni correction and has more power to detect true effects comparatively. Coefficients and 95% confidence intervals (CIs) are reported. Software for Statistics and Data Science (STATA; V.16.0) was used for these analyses.

## 3 Results

**Table 2** describes the participant demographics, baseline mental health scores, and behavioural problem scores. Notably, 41% of youth exhibited a high-risk baseline mental health status, indicating a considerable risk of clinically significant problems. Predominantly, youths allocated the most time (≥5 hours/day) to education-related screen time, while spending less time on video games (<3 hours/day) across all assessed periods. Pre-intervention, 38.4% of youth reported engaging in less video viewing screen time (<3 hours), a percentage that increased to 52.9% immediately post-intervention and further rose to 55.4% one month following the program.

**Table 2:** Mean score of behavioural variables at each survey time point and distribution of participant demographics at baseline.

	Pre- intervention (*n=*117)	Post- intervention (*n* =60)	Follow-up (*n* =51)
*Conduct Problems Score*	2.6 ± 2.3	2.5 ± 2.2	2.2 ± 2.0
*Hyperactivity Score*	6.2 ± 2.4	5.7 ± 2.4	5.3 ± 2.5
Baseline Mental Health Status			
Low Risk	31.3%		
Moderate Risk	27.8%		
High Risk	40.9%		
Age			
Adolescent	66.7%		
Young Adult	33.3%		
Sex			
Male	21.6%		
Female	78.4%		

Youth average conduct problem score was 2.6 ± 2.3 at baseline, classified within the low-risk range, and remained relatively unchanged at post and follow-up survey time points. In contrast, the average score for hyperactivity (6.2 ± 2.4), considered moderate risk for the significant problem (cut-off score = 6.15) at baseline, was improved after the mindfulness program at both post-survey (5.7 ± 2.4) and follow-up (5.3 ± 2.5) compared to the baseline score (6.2 ± 2.4). We examined the relationship between four types of screen time on each outcome variable (i.e., conduct problems and hyperactivity). **Table 3** shows that over 5 hours of playing video games is significantly associated with increased conduct problems [*β*= -1.75, 95% CI=-0.20 – 3.30, *p*=0.027]. However, after accounting for the multiple comparisons, the association between video games and conduct problems no longer had a p-value below 0.05 (*p*=0.08).

**Table 3:** Associations between behavoural problems (conduct problem and hyperactivity) and screen time (Coefficients^φ^, 95% confidence intervals) at baseline (pre-survey).

	Conduct problems	Hyperactivity
Video Viewing		
<1 hrs/day	Ref	Ref
1-3 hrs/day	1.24 (-0.81 to 3.29)	1.05 (-1.10 to 3.21)
3-5 hrs/day	1.01 (-1.07 to 3.10)	0.02 (-2.18 to 2.21)
>5 hrs/day	1.41 (-0.61 to 3.42)	0.87 (-1.26 to 2.99)
Social Media		
<1 hrs/day	Ref	Ref
1-3 hrs/day	-0.01 (-1.22 to 1.20)	-0.55 (-1.82 to 0.72)
3-5 hrs/day	-0.44 (-1.86 to 0.99)	0.00 (-1.50 to 1.50)
>5 hrs/day	0.34 (-0.93 to 1.62)	-0.58 (-1.92 to 0.76)
Video Gaming		
<1 hrs/day	Ref	Ref
1-3 hrs/day	0.40 (-0.64 to 1.43)	0.29 (-0.81 to 1.38)
3-5 hrs/day	0.48 (-0.83 to 1.78)	1.19 (-0.19 to 2.56)
>5 hrs/day	1.75 (0.20 to 3.30)* †	0.54 (-1.09 to 2.17)
Educational Screen Time		
<1 hrs/day	Ref	Ref
1-3 hrs/day	1.64 (-0.31 to 3.58)	1.44 (-0.61 to 3.48)
3-5 hrs/day	0.83 (-1.00 to 2.65)	0.29 (-1.64 to 2.21)
>5 hrs/day	1.13 (-2.75 to 2.69)	1.14 (-0.64 to 2.92)

* p<0.05; φ unstandardized; † no longer significant after correcting for multiple comparisons.

In a partially adjusted model (**Table 4**), accounting for age, sex and baseline mental health status, the mindfulness intervention program significantly contributed to decreased hyperactivity at time 2 (post-intervention) compared to the baseline [*β*=-0.54, 95% CI=-0.96 to -0.12, *p*=0.01], and it was maintained at follow up [*β*=-0.73, 95% CI=-1.30 to -0.15, *p*=0.01]. The impact of the mindfulness intervention program on hyperactivity remained significant even after accounting for screen time, indicating a beneficial effect of mindfulness intervention in reducing hyperactivity [post-interention: *β*=-0.49, 95% CI=-0.91 to -0.08, *p*=0.02; follow up: *β*=-0.64, 95% CI=-1.26 to -0.03, *p*=0.04]. This beneficial effect was shown immediately after the intervention, and it remained significant at time 3 (1 month past the completion of the mindfulness program) [*β*=-0.64, 95% CI=-1.26 to -0.02, *p*<.05]. These associations accounted for baseline mental health status and demographic factors (sex, age) as well, in addition to screen time (**Table 3**). Moderate and high-risk mental health status was associated with hyperactivity [*β*=1.58, 95% CI=0.69 to 2.47, *p*<0.01; *β*=-3.31, 95% CI=-2.48 to 4.13, *p*<0.001, respectively). Adjusted p-values for post- and follow-up surveys remained at p<.05 level (post: p=0.042, follow-up: p=0.045) after accounting for the multiple comparisons, indicating that mindfulness-based interventions may effectively decrease hyperactivity problems. No significant associations were found between the time points and conduct problems.

**Table 4:** Associations between behavioural problems (hyperactivity and conduct problems), screen time, and participant demographics (Coefficients†, 95% confidence intervals).

	Hyperactivity		Conduct problems	
	Partially adjusted	Fully adjusted	Partially adjusted	Fully adjusted
Time				
Pre	Ref	Ref	Ref	Ref
Post	-0.54 (-0.96 to -0.12)*	- 0.49 (-0.69 to 2.47)*	-0.02 (-0.39 to 0.35)	0.01 (-0.36 to 0.36)
Follow up	-0.73 (-1.30 to -0.15)*	-0.64 (-1.26 to -0.03)*	-0.24 (-0.61 to 0.13)	-0.17 (-0.55 to 0.22)
SDQ baseline				
Low Risk	Ref	Ref	Ref	Ref
Moderate Risk	1.65 (0.75 to 2.54)***	1.58 (0.69 to 2.47)**	0.81 (-0.0 to 1.67)*	0.85 (-0.04 to 1.75)
High Risk	3.41 (2.59 to 4.23)***	3.31 (2.48 to 4.13)***	2.67 (1.85 to 3.48)***	2.63 (1.80 to 3.46)***
Sex				
Female	Ref	Ref	Ref	Ref
Male	0.23 (-0.62 to 1.08)	0.20 (-0.70 to 1.10)	0.54 (-0.30 to 1.39)	0.63 (-0.25 to 1.50)
Age				
Adolescent	Ref	Ref	Ref	Ref
Young Adult	-0.71 (-1.44 to 0.03)	-0.09 (-0.18 to 0.01)	-0.88 (-1.62 to -0.15)*	-0.09 (-0.19 to 0.00)
Video Viewing				
<1 hrs/day		Ref		Ref
1-3 hrs/day		0.35 (-0.59 to 1.30)		-0.63 (-1.32 to 0.05)
3-5 hrs/day		-0.23 (-1.18 to 0.73)		-0.71 (-1.38 to -0.05)*
>5 hrs/day		-0.53 (-0.49 to 1.54)		-0.42 (-1.17 to 0.34)
Social Media				
<1 hrs/day		Ref		Ref
1-3 hrs/day		-0.32 (-1.02 to 0.37)		0.25 (-0.31 to 0.80)
3-5 hrs/day		-0.19 (-1.01 to 0.62)		0.02 (-0.61 to 0.64)
>5 hrs/day		0.08(-0.77 to 0.93)		0.40 (-0.31 to 1.12)
Video games				
<1 hrs/day		Ref		Ref
1-3 hrs/day		0.10 (-0.50 to 0.74)		-0.25 (-0.76 to 0.25)
3-5 hrs/day		0.57 (-0.20 to 1.36)		0.01 (-0.63 to 0.64)
>5 hrs/day		0.21 (-0.69 to 1.11)		0.40 (-0.31 to 1.12)
Educational				
<1 hrs/day		Ref		Ref
1-3 hrs/day		0.29(-0.64 to 1.22)		-0.38 (-1.02 to 0.26)
3-5 hrs/day		-0.04(-0.97 to 0.89)		0.13 (-0.56 to 0.82)
>5 hrs/day		0.05 (-0.77 to 0.87)		0.14 (-0.46 to 0.74)

* <.05, ** <.01, *** <.001; † unstandardized.

## 4 Discussion

This study addressed a current research gap by examining the efficacy of a mindfulness-based intervention in reducing hyperactivity and conduct problems among community youth while accounting for four types of screen time. Our findings indicated the mindfulness-based intervention program was significantly associated with reduced hyperactivity with and without accounting for demographic covariates.

Our findings align with the previous literature that suggests a positive association between video gaming and conduct problems (10, 13, 14, 16). Due to the secondary analysis of an existing dataset, we were limited to examining the specificity of the video games (e.g., degree of violence) that may be associated with increased conduct problems. Further, after adjusting the p-values accounting for multiple comparisons, the association between video game and conduct problems were no longer significant. Hence, due to the limitations of our study, this finding may need to be taken with caution, and the result warrants further replication to ascertain its impact. Individuals with hyperactivity are challenged with delaying gratification (42). Video games may provide instant gratification, which may underlie this association between video games and increased behavioural problems. Unlike previous studies (15, 17), we didn’t find a significant association between screen time and hyperactivity. We suspect that the older age range of the participants (graduate students) and using an average time spent on a screen across five different activities (17) may have contributed to the different findings. Likewise, while we used one domain of ADHD symptoms (i.e., hyperactivity), previous meta-analysis mostly included studies that used a composite score of ADHD symptoms encompassing inattention, hyperactivity and impulsivity (15).

Our findings suggest that a 12-week online mindfulness program is significantly associated with reduced hyperactivity. Its effect remained significant four weeks after program completion while accounting for screen time usage. Our results confirm previous studies that found a positive impact of mindfulness in reducing hyperactivity and suggested that mindfulness may serve as a non-medicinal intervention in managing challenges with impulse control and reactivity (20, 24). Our findings align with previous notions that longer duration (i.e., > seven weeks) of MBIs might be more effective in reducing hyperactivity (20).

However, the mindfulness intervention studied here was not significantly associated with reduced conduct problems among youth. While the relationship between mindfulness and conduct problems is vastly understudied, our finding does not align with a study that tested the efficacy of mindfulness programs on adolescents with conduct disorders in Indonesia (43). The authors of that study found that mindfulness was effective in reducing antisocial behaviour and observed a reduction in serum cortisol. There are a few discrepancies between this and the present study, though. For example, Dewi, Wiwie (43) only included male adolescents, while the majority (78.4%) of our sample consisted of females. Besides, our participants were community youth with a low-risk range of conduct problems at baseline. The underlying mechanism and symptom manifestation of conduct problems/psychopathy may differ between males and females (44), which may explain the discrepancy in the findings. We speculate that the heterogeneous conduct problems make it difficult for mindfulness programs to provide targeted support. Also, mindfulness may help mitigate exacerbated conduct problems but has limited impact on improving those with a low-risk range of conduct problems. Future studies are warranted to clarify the efficacy of mindfulness programs on conduct problems.

There are several limitations to our study. Primarily, due to recruitment challenges during the pandemic, participants were predominantly located in a rural region. Additionally, the sample exhibited a lack of heterogeneity in sex and gender distribution, with the majority being females. This constraint restricts the generalizability of our findings. To ensure broader applicability across the youth population, future studies should strive for a more balanced sex ratio and diverse representation in terms of geographical location. Second, as we conducted a secondary analysis, we could not explore the nature of video games in detail (e.g., whether they were violent and aggressive); therefore, our results do not offer in-depth insight into which aspects of video gaming are associated with adverse outcomes. Third, this study has a simple pre-post design using self-reports without utilizing a control group, which limits the certainty that the observed changes over time result from the intervention and presents a risk for measuring bias. Lastly, almost half of the participants did not complete post-intervention and follow-up surveys, resulting in a relatively low sample size and large attrition rate. To adjust for the retention issue, we applied GLS via the Maximum Likelihood (ML) model to account for missing data and an unstructured model in consideration of the small sample size and less than 5 time points (40). Future studies should use representative samples with a more nuanced assessment of electronic device use to better understand the mediators and moderators of the associations between screen time and behavioural problems.

## 5 Conclusion and clinical implications

Despite these limitations, the results of the present study suggest that non-medicinal intervention programs, such as mindfulness-based interventions, can effectively manage hyperactivity. Given the relatively high prevalence of hyperactivity (45, 46) and its pharmacological treatment (4.5% in North America) (47), this outcome is of utmost importance. The side effects of psychotropic medications are well-known (46, 48), which underscores the relevance of alternative, safer treatment options. Considering the simplicity, brevity (and so cost-effectiveness), non-invasive nature and other mental health benefits of the mindfulness intervention studied here (33, 49), we argue that the results are quite promising and worthy of further study and larger-scale implementation. Further, our data reflect youth’s current communication that is inherently embedded in internet-based technology/social media, which has integrated into our daily lives and social lifestyle and heightened screen time trends during the pandemic. It was not until the late 2010s that handheld devices such as smartphones and tablets became prevalent among youth, allowing them to access and connect through those devices ubiquitously.

## Data availability statement

The data supporting the conclusions of this article will be made available by the authors, without undue reservation.

## Ethics statement

The studies involving humans were approved by Waypoint Centre for Mental Healthcare. The studies were conducted in accordance with the local legislation and institutional requirements. Participants consented to participate. For youth who were younger than 18 years old, an information letter was provided to the parents/guardians and written informed consent for participation in this study was provided by the participants’ legal guardians/next of kin.

## Author contributions

SK conceived and designed the study. SK and SM managed the dataset and conducted the statistical analysis. All authors discussed and interpreted the results. SK drafted the manuscript, and all authors provided critical reviews and contributed to the final manuscript. All authors contributed to the article and approved the submitted version.
